# Interpretation of Chemical Profiles in Terms of Food Quality, Safety and Authenticity: Advancing Foodomics Approaches

**DOI:** 10.3390/foods15213924

**Published:** 2026-11-02

**Authors:** Laura Alessandroni, Simone Angeloni

**Affiliations:** Chemistry Interdisciplinary Project (ChIP), School of Pharmacy, University of Camerino, 62032 Camerino, Italy; simone.angeloni@unicam.it

## 1. Introduction

The rapid evolution of high-resolution mass spectrometry, next-generation sequencing, and multi-omics platforms has shifted food analysis from monitoring individual markers to untargeted analysis of all analyzable compounds, with the aim of decoding multi-layered molecular ecosystems. In this context, foodomics integrates advanced analytical platforms with chemometrics and bioinformatics approaches to translate structural complexity and chemical characterization into parameters that can be used to assess food quality, safety, authenticity, and functionality [1,2,3].

The Special Issue entitled “Applications of Foodomics Strategies in Advancing Food Quality, Safety and Authenticity” was conceived within this context, with the aim of highlighting recent applications of foodomics across different food matrices and analytical challenges. The Special Issue includes five contributions addressing dairy products, coffee, fermented beverages, and agro-industrial byproducts. Three contributions provide comprehensive reviews of emerging foodomics applications, while two original research articles demonstrate the application of untargeted mass spectrometry and artificial intelligence to specific food-related questions.

Although diverse in terms of matrices, analytical platforms, and scientific objectives, the contributions collected in this Special Issue share a common perspective: foodomics is increasingly moving from the generation of complex molecular fingerprints toward the integration and interpretation of multidimensional datasets. This transition is essential to provide information that can be translated into robust tools for food quality control, safety assessment, authenticity verification, and the valorization of food resources.

## 2. Contributions to the Special Issue

Addressing complex dairy systems, Xu et al. established a multi-omics blueprint integrating proteomics, metabolomics, lipidomics, and metagenomics to advance nutritional profiling, adulteration detection, and predictive shelf-life modeling. Shifting to complex botanical matrices, Wang et al. highlighted mass spectrometry-based lipidomics in coffee, emphasizing how lipid transformations govern aroma release and flavor development while highlighting current gaps in sensory mechanism mapping. Complementing this analytical focus, Felizzato et al. evaluated coffee origin and aroma intensity by coupling HS-SPME-GC-MS with explainable artificial intelligence (SHAP), demonstrating how computational tools can extract interpretable molecular drivers beyond simple classification. In the domain of complex fermentations, Song et al. applied a systems biology framework to Baijiu, a typical Chinese liquor, integrating multi-omics layers to untangle microbe–metabolite networks and metabolic pathways. Finally, addressing resource circularity, Alessandroni et al. mapped the chemical profile of olive mill wastewaters via untargeted UHPLC-QTOF-MS, proving that foodomics workflows can effectively unlock high-value bioactive compounds from agro-industrial byproducts.

Beyond their specific matrix applications, these five studies collectively exposed the potential and weaknesses of foodomics. They addressed aspects ranging from the characterization of nutritional and safety properties to the assessment of authenticity, sensory quality, fermentation processes, and the valorization of food byproducts.

## 3. From Foodomics Applications to Common Challenges

Despite the diversity of the contributions, several common challenges emerge when they are evaluated together. Among these, the fundamental need for standardization and reproducibility stands out. Actually, foodomic workflows involve multiple stages, including sampling, sample preparation, instrumental acquisition, data processing, statistical analysis, and biological interpretation. Variability introduced at any of these stages can influence the final results. The need for standardized procedures remains one of the most important challenges that foodomics will have to face. The requirement of minimum reporting standards and harmonized procedures has been recognized since the establishment of the Metabolomics Standards Initiative and remains highly relevant to contemporary foodomics [4,5]. More consistent reporting of experimental conditions, quality-control procedures, data-processing parameters, and metadata, along with the availability of raw data, would facilitate the interpretation, comparison, and reproducibility of foodomics studies.

Equally critical is the challenge of confirming the discovered biomarker. In fact, untargeted metabolomics and other foodomics approaches are particularly effective for discovering candidate markers; however, identifying a discriminatory feature in a single dataset does not guarantee its applicability to all samples of that matrix. Differences in geographical origin, cultivar or species, production year, environmental conditions, processing, and storage can all affect food molecular profiles. Therefore, as highlighted by recent reviews on untargeted metabolomics for food authentication, it is important to move beyond marker discovery to their confirmation and validation through the application of targeted analytical methods [6,7].

Beyond candidate identification, transitioning from statistical correlation to mechanistic understanding represents another essential hurdle. Foodomic datasets can reveal strong statistical associations between molecular features and food attributes, but correlations do not demonstrate causality. This is particularly relevant when attempting to relate molecular profiles to sensory characteristics, biological activity, or technological properties. Future studies should therefore increasingly combine foodomics with controlled experiments, targeted quantitative analyses, sensory evaluation, microbiological investigations, and mechanistic approaches.

In parallel, the expanding role of machine learning and artificial intelligence introduces both transformative possibilities and methodological risks. Computational approaches can extract complex patterns from high-dimensional foodomics datasets and improve classification or prediction. However, model performance is highly dependent on dataset size and representativeness, feature selection, appropriate validation strategies, and the prevention of overfitting. The development of explainable approaches is particularly relevant when foodomic models are intended to support scientific interpretation or practical decision-making. Deep learning approaches have already demonstrated considerable potential in food analysis, but their broader application requires careful consideration of data quality, model interpretability, and external validation [8].

Ultimately, a persistent gap separates research-scale foodomics from industrial and routine implementation. The sophisticated analytical workflows used in research laboratories may not always be compatible with the requirements of industrial or regulatory applications in terms of speed, cost, robustness, or standardization. Bridging this gap will require not only technological improvements but also the development of validated workflows, reference materials, shared databases, and analytical strategies that can be transferred seamlessly between laboratories and food systems.

## 4. Future Research Directions

The mentioned challenges point toward several priority research directions that will shape the next generation of foodomics:

- Integrated multi-omics approaches: Future studies should combine metabolomics and lipidomics with proteomics, genomics, metagenomics, volatilomics, and spectroscopic techniques. The primary methodological goal is not merely aggregating larger datasets, but developing computational strategies capable of linking these heterogeneous molecular layers in a statistically robust and biologically meaningful manner [9].
- Enhanced metadata and data interoperability: Expanding the availability of standardized, publicly accessible databases enriched with comprehensive metadata—including cultivar, geographical origin, agricultural practices, processing history, and sensory attributes—is essential to support external model validation and long-term reproducibility.
- Bridging untargeted discovery and targeted validation: While untargeted workflows excel at hypothesis generation, translating candidate biomarkers into practical tools requires establishing optimized pipelines capable of moving directly from feature identification to targeted quantitative analysis validation.
- Explainable artificial intelligence: Moving beyond black-box classification, machine learning frameworks should increase explainability to directly connect molecular features with functional, sensory, and biological traits, backed by transparent reporting of model uncertainty.
- At-line and real-time monitoring: Coupling high-throughput or miniaturized analytical tools with automated processing pipelines will facilitate point-of-need monitoring along the supply chain, shifting foodomics from retrospective laboratory analysis to proactive process control.
- Circular economy and side-stream valorization: As demonstrated by olive mill wastewater profiling, foodomics can accelerate resource recovery. Future research must integrate molecular characterization with extraction efficiency, safety profiles, techno-economic feasibility, and life-cycle assessments to ensure that proposed valorization routes are genuinely sustainable.

## 5. Conclusions

The five contributions collected in this Special Issue illustrated the broad potential of foodomics in advancing the characterization and understanding of food systems. In particular, it has been noted that multi-omics approaches provide increasingly comprehensive information on complex food matrices; lipidomics and volatile profiling can contribute to the investigation of quality and sensory properties; systems biology can reveal interactions within fermentation ecosystems; explainable artificial intelligence can facilitate the interpretation of complex analytical datasets; and untargeted metabolomics can reveal new opportunities for the valorization of food-processing side streams.

At the same time, the contributions highlight that the future progress of foodomics will depend less on the ability to generate increasingly large datasets and more on the ability to validate, integrate, interpret, and translate those datasets into reliable knowledge.

Standardization, reproducibility, biomarker validation, external model validation, explainable artificial intelligence, and interoperable databases will therefore remain central priorities. Equally important will be the development of stronger connections between exploratory foodomics and targeted analytical validation, as well as closer collaboration among food chemists, food technologists, microbiologists, sensory scientists, data scientists, industry, and regulatory stakeholders.

Ultimately, bridging the gap between exploratory data acquisition and routine quality assurance requires not larger datasets, but rather better-validated workflows. By transforming complex molecular fingerprints into reliable, interpretable knowledge, foodomics can solidify its role as a core analytical driver for a safer, more transparent, and sustainable food supply chain.
